# O-GlcNAcylation is crucial for sympathetic neuron development, maintenance, functionality and contributes to peripheral neuropathy

**DOI:** 10.3389/fnins.2023.1137847

**Published:** 2023-05-05

**Authors:** Hsueh-Fu Wu, Chia-Wei Huang, Jennifer Art, Hong-Xiang Liu, Gerald W. Hart, Nadja Zeltner

**Affiliations:** ^1^Center for Molecular Medicine, University of Georgia, Athens, GA, United States; ^2^Department of Biochemistry and Molecular Biology, University of Georgia, Athens, GA, United States; ^3^Complex Carbohydrate Research Center, University of Georgia, Athens, GA, United States; ^4^Biomedical and Translational Sciences Institute, Neuroscience Program, University of Georgia, Athens, GA, United States; ^5^Regenerative Bioscience Center, Department of Animal and Dairy Science, College of Agricultural and Environmental Sciences, University of Georgia, Athens, GA, United States; ^6^Department of Cellular Biology, University of Georgia, Athens, GA, United States

**Keywords:** O-GlcNAcylation, peripheral nervous system, autonomic nervous system, sympathetic neuron, human pluripotent stem cells, diabetes, hyperglycemia, peripheral neuropathy

## Abstract

O-GlcNAcylation is a post-translational modification (PTM) that regulates a wide range of cellular functions and has been associated with multiple metabolic diseases in various organs. The sympathetic nervous system (SNS) is the efferent portion of the autonomic nervous system that regulates metabolism of almost all organs in the body. How much the development and functionality of the SNS are influenced by O-GlcNAcylation, as well as how such regulation could contribute to sympathetic neuron (symN)-related neuropathy in diseased states, remains unknown. Here, we assessed the level of protein O-GlcNAcylation at various stages of symN development, using a human pluripotent stem cell (hPSC)-based symN differentiation paradigm. We found that pharmacological disruption of O-GlcNAcylation impaired both the growth and survival of hPSC-derived symNs. In the high glucose condition that mimics hyperglycemia, hPSC-derived symNs were hyperactive, and their regenerative capacity was impaired, which resembled typical neuronal defects in patients and animal models of diabetes mellitus. Using this model of sympathetic neuropathy, we discovered that O-GlcNAcylation increased in symNs under high glucose, which lead to hyperactivity. Pharmacological inhibition of O-GlcNAcylation rescued high glucose-induced symN hyperactivity and cell stress. This framework provides the first insight into the roles of O-GlcNAcylation in both healthy and diseased human symNs and may be used as a platform for therapeutic studies.

## Introduction

O-GlcNAcylation is a monosaccharide post-translational modification (PTM), in which a single sugar, O-linked N-acetylglucosamine (O-GlcNAc), is added onto Serine/Threonine residues of nuclear, cytosolic, and mitochondrial proteins. O-GlcNAcylation is dynamic, reversible, abundant, and thus it is involved in regulating nearly all kinds of key cellular processes, including transcription, translation, signal transduction, and energy homeostasis (Hart, 2019). O-GlcNAcylation is responsive to nutrient state and has been found essential for neural development (Sheikh et al., 2021). Global O-GlcNAc levels decrease during neural differentiation in both stem cell and animal models (Sheikh et al., 2021). Elevating protein O-GlcNAcylation using genetic or pharmacological methods disturbs mouse brain development and cortical neuron differentiation from embryonic stem cells (Olivier-Van Stichelen et al., 2017; Parween et al., 2017). O-GlcNAcylation also plays critical roles in neurological disorders. In Alzheimer’s disease for example, decreased O-GlcNAcylation has been found and linked to tau and amyloid-β peptide pathology (Yuzwa and Vocadlo, 2014; Zhu et al., 2014; Huang et al., 2023). Similarly, dysregulated O-GlcNAcylation is associated with synucleinopathies in Parkinson’s disease (Balana and Pratt, 2021).

The majority of current studies of O-GlcNAcylation functions on neural development are focusing on the central nervous system (CNS). Its roles in peripheral nervous system (PNS) functions and diseases, in contrast, are vastly understudied. The sympathetic nervous system (SNS) belongs to the autonomic nervous system of the PNS. SNS innervates almost all body organs, such as heart, blood vessels, adipose tissues, lung, kidney, and stomach, and thus controls body homeostasis without consciousness by modulating heart rate, blood pressure, blood glucose, and gland secretion (Scott-Solomon et al., 2021). Dysregulation of the SNS has been seen in various metabolic diseases, particularly cardiovascular complications and diabetes (Tentolouris et al., 2008; Zhang and Anderson, 2014; Lambert et al., 2015; Scott-Solomon et al., 2021). SNS hypersensitivity characterized by over-released norepinephrine (NE) and sympathetic nerve hyperactivity was identified in patients and mouse models of hypertension (Fisher and Paton, 2012; Li et al., 2012; Shanks et al., 2013; DeLalio et al., 2020). Increased sympathetic tone as well as NE levels have been reported in both type I and type II diabetes together with the metabolic syndrome, and therefore was suggested as a key diagnostic assessment for an early characterization strategy (Lambert et al., 2015). Interestingly, increased protein O-GlcNAcylation has also been found in these disorders (Lima et al., 2009; Ma and Hart, 2013).

Several studies have revealed the importance of O-GlcNAcylation in peripheral cell lineages: Kim et al. showed that O-GlcNAcylation in Schwann cells (SCs) is required for functional myelination and axonal integrity (Kim et al., 2016, 2018). Su et al. found that sensory neuron-specific O-GlcNAcylation disruption in mice causes cell loss and impaired axonal development (Su and Schwarz, 2017). While extensive studies have shown defects in symN target tissues, such as heart and blood vessels, and in metabolic diseases due to O-GlcNAcylation dysregulation (Lunde et al., 2012; Erickson et al., 2013; Silva-Aguiar et al., 2018; Masaki et al., 2020; Prisco et al., 2020; Hegyi et al., 2021; Umapathi et al., 2021), no study has been performed to understand the symN pathology itself, despite the SNS being the main system to control the metabolism of these tissues. Therefore, we hypothesized that O-GlcNAcylation may also play indispensable roles in symNs, both in healthy and diseased states.

The SNS consists of two neural systems: The preganglionic symNs, which anatomically belongs to the motor neuron system of the CNS and the postganglionic symNs, the neuron type that is actually located in the periphery and innervates the organs, as well as regulates their functions by secreting NE (Scott-Solomon et al., 2021). Studying postganglionic symN function in patients or animals is challenging since it is hard to exclude the input from the CNS. Alternatively, primary cultures from animal models provide another framework for isolated cell investigations. However, obtaining large enough cell numbers from patients and healthy controls, as well as general differences between human and non-human cells remains a serious limitation (Mestas and Hughes, 2004; Bracken, 2009). Hence, hPSC-derived neurons provide a promising and unlimited cell source model system to overcome these challenges (Zhu and Huangfu, 2013; Saito-Diaz and Zeltner, 2019).

SNS dysfunction in diabetes that contributes to cardiovascular complications has been studied previously in animal models and patients. Disease symptoms initiate with sympathetic hyperactivity and NE spillover (Kribben and Philipp, 1989; Kaul, 1999; Perin et al., 2001), and gradually worsen by sympathetic denervation and reduced NE release, which is known as diabetic autonomic neuropathy (DAN) (Schmidt, 1996, 2002a,b; Vinik et al., 2003). In type 1 diabetes (T1D), sympathetic overactivity has been proposed as the primary mechanism that leads to hypertension, or as the cause of chronic renal failure through the renin-angiotensin-aldosterone system (RAAS) (Perin et al., 2001). Noticeably, the RAAS system is also present in postganglionic symNs (Bardsley et al., 2020; Wu et al., 2022), suggesting that intrinsic metabolic dysregulation of symNs might be one of the main mechanisms underlying neuropathy in T1D. Likewise, in type 2 diabetes (T2D), sympathetic overactivity is also observed at the time when diabetes is diagnosed, along with abnormal glucose tolerance and insulin-resistance (Perin et al., 2001). The excessive sympathetic tone coupled with impaired adrenergic signaling might be a reason of insulin-resistance and cardiovascular disability (Perin et al., 2001; Thaung et al., 2015). On the other hand, the axonal degeneration and dystrophy of neural terminals (Schmidt, 1996) in diabetes that leads to DAN are highly selective to the autonomic ganglia, specifically the postganglionic symNs (Schmidt, 2002a,b). The impaired neural transmission between postganglionic symN axons and targeted tissues is likely to determine the progress of DAN, which is age-related (Schmidt, 2002a,b; Campbell-Thompson et al., 2021). Accordingly, functional defects in postganglionic symNs evident by altered dendritic structures as well as axonal elements are suggested as the cause of dysautonomia in diabetes (Schmidt et al., 1993, 2008). This evidence provides solid observations of symN-specific dysfunction among the progress of diabetic neuropathy, and therefore make diabetes an ideal disease model to study the role of O-GlcNAcylation in symN dysfunction.

In this study, we assessed O-GlcNAcylation levels during symN development and maintenance using a well-established postganglionic symN differentiation protocol (Wu and Zeltner, 2020; Wu et al., 2022). We showed that O-GlcNAcylation was stably present through the entire lifespan of symN development and was required for symN growth and survival. We also established a hyperglycemia model, which might recapitulate intrinsic symN hyperactivity as an early indication of SNS dysfunction in diabetes. Additionally, we demonstrated that high glucose-induced O-GlcNAcylation in symNs is responsible for the hyperactivity phenotype. Our results unveil the regulatory roles of O-GlcNAcylation in peripheral symNs, and the human based model system might be used as a platform for therapeutic discovery in the future.

## Materials and methods

### Pluripotent stem cells

Embryonic stem cell WA09 (H9) line was used in this study. Cells were maintained in Essential 8 medium (Gibco, A15170-01) and vitronectin coated (Thermo Fisher/Life Technologies, A14700, 5 μg/ml) cell culture plates. For passaging, cells were dissociated using EDTA (Sigma, ED2SS).

### Sympathetic neuron differentiation

A detailed differentiation protocol (Wu and Zeltner, 2020) and an optimized protocol (Wu et al., 2022) with freezing method can be found in our previous studies. Briefly, hPSCs are dissociated by EDTA and replated in day 0–1 medium containing Essential 6 medium (Gibco, A15165-01), 0.4 ng/ml BMP4 (PeproTech, 314-BP), 10 μM SB431542 (R&D Systems, 1,614) and 300 nM CHIR99021 (R&D Systems, 4,423) on Geltrex (Invitrogen, A1413202) coated cell culture plates at 1.25×10^5^/cm^2^ density. On day 2, the cells were fed with day 2–10 medium containing Essential 6 medium, 10 μM SB431542 and 0.75 μM CHIR99021. On day 10, NCCs were dissociated by Accutase (Coring, AT104500) and aggregated on ultra-low attachment plates (Corning, 07200) in day 10–14 medium containing Neurobasal medium (Gibco, 21103-049), B27 (Gibco, 17502-048), L-Glutamine (Thermo Fisher/Gibco, 25030-081), 3 μM CHIR99021 and 10 ng/ml FGF2 (R&D Systems, 233-FB/*CF*). On day 14, sympathetic neuroblast spheroids are dissociated by Accutase and replated on PO (Sigma, P3655)/LM (R&D Systems, 3400-010-01)/FN (VWR/Corning, 47743-654) coated plates at 1×10^5^/cm^2^ density in symN medium containing Neurobasal medium, B27, L-Glutamine, 25 ng/ml GDNF (PeproTech, 450), 25 ng/ml BDNF (R&D Systems, 248-BD), 25 ng/ml NGF (PeproTech, 450-01), 200 μM ascorbic acid (Sigma, A8960), 0.2 mM dbcAMP (Sigma, D0627) and 0.125 μM retinoic acid (Sigma, R2625, add retinoic acid freshly every feeding). To enhance purity, 0.5 μM aphidicolin (Cayman, 14007) can be added to the symN medium from day 20–30. Please note that BMP4 quality is highly lot-dependent and therefore a titration test of BMP4 concentration for each batch of BMP4 is highly recommended.

### Hyperglycemia model

**Figure 1:**
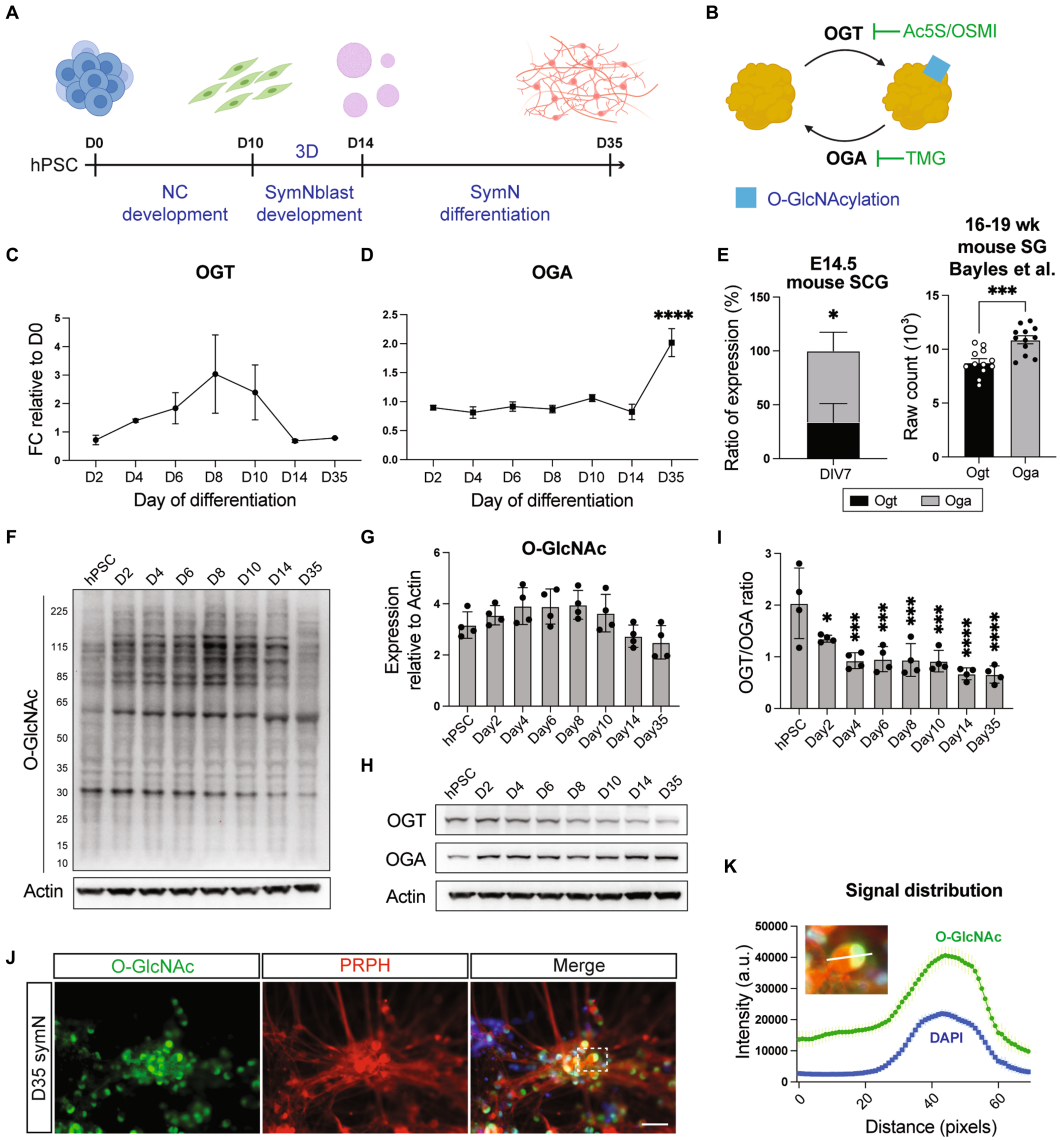
High glucose environment leads to symN hyperactivity. **(A)** Schematic illustration of high glucose treatments of symNs. **(B)** Multielectrode array (MEA) analysis of symN activity over time. Two-way ANOVA followed by Dunnett’s multiple comparisons. *n* = 4 biological replicates. **(C)** Representative heatmap of the spike rate on day 45. **(D)** Representative spike map on day 45. Black bars represent individual spikes. Blue bars represent bursts. **(E)** Representative immunofluorescent staining for c-Fos and PRPH on day 45. Scale bar = 50 μm. **(F)** Quantification of c-Fos intensity. Unpaired *t*-test. *n* = 3 biological replicates. *p < 0.05. Error bars represent SEM.

In all experiments in this study that challenged symNs with high glucose (30 mM), including the glucose titration test (Figure 1), symNs were first preconditioned in the medium that contained 5 mM glucose for 5 days, and then switched to the desired high glucose concentrations for five more days. To prevent the effect of changed osmotic pressure, non-metabolizable mannitol (Sigma, M4125) was added during the entire treatment as previously described (Pekkurnaz et al., 2014).

### Mouse SCG primary culture

SCG tissues from WT C57BL/6 mice were provided by Dr. Hong-Xiang Liu’s lab at University of Georgia. The use of animals was approved by the Institutional Animal Care and Use Committee at the University of Georgia. The experiment was performed following the National Institutes of Health Guidelines for the care and use of animals in research. The mouse SCGs at E14.5 were dissociated and cultured using previously described protocols (Ch'ng et al., 2005; George et al., 2013).

### Microfluidic chamber culture

The culture device was purchased from eNUVIO. SymNs were plated on one side of the culture device in the “cell body” chamber on day 14. SymN medium was fed equally to both sides until day 20, and from then on the cell body chamber was fed with symN medium containing no NGF at low volume, while the other side was fed with symN medium containing NGF at high volume to create the NGF gradient. Axons across the microfluidic tunnels can been clearly observed on day 30 on the side defined as the “axon” chamber, from which point on the medium on both sides was changed to 5 mM glucose medium for 5 days. On day 35, symNs were fed with 5 mM or 30 mM medium for 5 more days. On day 40, axons were trimmed using suction with a micropipette tip, and the level of regeneration was assessed after 48 h.

### RT-qPCR

0.5×10^6^ cells of the cell lysates were collected using Trizol (Invitrogen, 15596026) for each sample. Reverse transcription was performed with 1 μg total RNA using iScript^™^ Reverse Transcription Supermix (Bio-Rad, 170884). SYBR green (Bio-Rad) RT-qPCR was performed using CFX96 Touch Deep Well Real-Time PCR Detection System (Bio-Rad) and analyzed by CFX Maestro. For OGT and OGA mRNA expression in mouse SCGs, we compared the expression level by taking the percentage of the ratio of 2^(−ΔCt)^. Primers were listed in Table 1.

**Table 1:** RT-qPCR primers used in this study.

Primers	Target	Catalog no.	Method	Sequence	
OGT	Human	Origene (NM_181672)	SYBR	Forward	CAGGAAGGCTATTGCTGAGAGG
				Reverse	CGGAACTCACATATCCTACACGC
OGA	Human	Origene (NM_012215)	SYBR	Forward	GCAAGAGTTTGGTGTGCCTCATC
				Reverse	GTGCTGCAACTAAAGGAGTCCC
Ogt_mouse	Mouse	Origene (NM_139144)	SYBR	Forward	GGCTATGTGAGTTCTGACTTCGG
				Reverse	GATTGGCTTCCGCCATCACCTT
Oga_mouse	Mouse	Origene (NM_023799)	SYBR	Forward	ACTGTGCCAACAGGACCATCCT
				Reverse	CTGTGCTGAAGAGTGACTACGAC
PHOX2B	Human	Frith et al., 2018	SYBR	Forward	CTACCCCGACATCTACACTCG
				Reverse	CTCCTGCTTGCGAAACTTG
ASCL1	Human	Origene (NM_004316)	SYBR	Forward	TCTCATCCTACTCGTCGGACGA
				Reverse	CTGCTTCCAAAGTCCATTCGCAC
TH	Human	Origene (NM_199292)	SYBR	Forward	GCTGGACAAGTGTCATCACCTG
				Reverse	CCTGTACTGGAAGGCGATCTCA
DBH	Human	Origene (NM_000787)	SYBR	Forward	GCCTTCATCCTCACTGGCTACT
				Reverse	CAGCACTGTGACCACCTTTCTC
PRPH	Human	Homemade	SYBR	Forward	GTGCCCGTCCATTCTTTTGC
				Reverse	GTCACCACCTCCCCATTCCG
SYP	Human	Origene (NM_003179)	SYBR	Forward	TCGGCTTTGTGAAGGTGCTGCA
				Reverse	TCACTCTCGGTCTTGTTGGCAC
GAP43	Human	Origene (NM_002045)	SYBR	Forward	GAGCAGCCAAGCTGAAGAGAAC
				Reverse	GCCATTTCTTAGAGTTCAGGCATG
MAP2	Human	Origene (NM_002374)	SYBR	Forward	AGGCTGTAGCAGTCCTGAAAGG
				Reverse	CTTCCTCCACTGTGACAGTCTG
GAPDH	Human	Origene (NM_002046)	SYBR	Forward	GTCTCCTCTGACTTCAACAGCG
				Reverse	ACCACCCTGTTGCTGTAGCCAA

### Western blot

Day 35 symNs in one well of 6-well culture plate (Corning) were prepared for protein lysate collection. SymNs were lysed by lysis solution that contains 1x RIPA buffer (Sigma-Aldrich, R0278), 1 mM PMSF protease inhibitor (Thermo Fisher Scientific, 36978), PhosSTOP phosphatase inhibitor (Sigma-Aldrich, 4906845001) and 10 μM PUGNAc (Sigma, A7229). Bradford reagent (Bio-Rad, 5000006) was used for measuring protein concentration. For western blotting, 20 μg/well of total protein for each sample was prepared and ran in 4–12% pre-cast Bis-Tris gels. Proteins were then transferred to nitrocellulose membrane and blocked with 5% BSA in TBST. The membranes were incubated with Anti-O-GlcNAc (1:1000, Cell Signaling, 82332), Anti- OGT (1:1000, home-made), Anti-OGA (1:1000, home-made), Anti-Milton1 (1:800, Proteintech, 13987-1-AP) Anti-Actin (1,5000, BD Pharmingen, 612656) primary antibodies at 4°C overnight. The next day, the membranes were washed 3 times with PBST and incubated with goat anti-mouse and goat anti- rabbit HRP at room temperature (RT) for 1 h.

### Wheat-germ agglutinin affinity isolation (pull-down assay)

O-GlcNAcylated proteins were pulled down with succinylated wheat-germ agglutinin (sWGA) coated agarose beads (Vector Laboratories, AL-1023S). Briefly, cell lysates were incubated with sWGA coated agarose beads overnight at 4 degrees. After incubation, the beads were collected by centrifugation at 200 g and washed three times with wash buffer (20 mM Tris–HCl pH 7, 20 mM NaCl, 0.1 mM EDTA, 1 mM PUGNAc, 1% TritonX-100, 1X protease inhibitor). Bound proteins were separated from beads by boiling with 2x laemmli buffer, and the isolated O-GlcNAcylated proteins were detected using western blotting as mentioned above.

### Immunofluorescent staining

SymNs were fixed by 4% paraformaldehyde (Sigma) for 20 min and washed twice with 1x PBS. Fixed symNs were permeabilized using 0.3% Triton, 1% BSA and 3% goat or donkey serum in 1x PBS for 20 min. Anti-O-GlcNAc (1:200, Cell Signaling, 82332; 1:200, home-made, CTD110.6 and RL2), Anti-PRPH (1:500, Santa Cruz, SC-377093), Anti-c-Fos (1:200, Santa Cruz, sc-166940), Anti-OGT (1:200, home-made) primary antibodies were added to the cells and incubated overnight at 4°C. The next day, cells were washed twice and incubated with secondary antibodies for 1 h at RT. Images were taken using the Lionheart FX Automated Microscope.

### Mitochondria tracing

Mitochondria staining of fixed neurons was performed using MitoView^™^ Green (Biotium, 70054) as the manufacturer’s instruction. Briefly, fixed symNs were incubated with 100 nM dye solution in PBS at RT for 15 min. For co-staining with regular immunofluorescent stain, neurons were stained with MitoView afterward. After staining neurons were imaged using the ECHO Revolve Microscope.

### Multielectrode array

Detailed methods of symN activity measurements were performed as described previously (Wu et al., 2022). SymNs were plated on PO/LM/FN coated MEA plates (Axion BioSystems, BioCircuit) and measured using a MEA plate reader (Axion BioSystems, Maestro Pro) under the neural detection mode according to manufacturer’s instruction.

### ROS assay

Live symN labeling using CM-H2DCFDA (Invitrogen, C6827) was performed according to manufacturer’s instruction. Briefly, symNs were incubated with 10 mM CM-H2DCFDA in PBS for 45 min at 37°C. After the incubation, GFP signal was measured using the Fluoroskan Ascent^™^ FL Microplate Fluorometer (ThermoFisher) at 492 nm excitation and 520 nm emission.

### Mitochondrial membrane potential

Live symN labeling using JC-1 (MedChemExpress, HY-15534) was performed according to manufacturer’s instruction. Briefly, symNs were incubated with 2 μM JC-1 in PBS for 30 min at 37°C. After the incubation, the ratio of RFP/GFP signal was measured using the Fluoroskan Ascent^™^ FL Microplate Fluorometer (ThermoFisher).

### MTT assay

SymN viability was measured using the PrestoBlue Viability Reagent (Invitrogen, A13261) according to manufacturer’s instruction. Briefly, medium of symNs on the desired timepoint was changed with 1:10 diluted viability reagent with fresh culture medium, and incubated for 3 h at 37°C. After that, 100 μl of medium and dye mixture of each well was taken and measured using a microplate reader (BioTek) immediately at 570 and 600 nm absorbance.

### Statistical analysis

At least three independent experiments (biological replicates) were collected for statistical analysis. Biological replicates (Chan and Teo, 2020) are defined as independent experiments performed several days apart either started from a new frozen vial of that particular cell line (Wong et al., 2017), or from a consecutive passage number of that cell line. Statistical analysis is described within individual figures/figure legends. Analyses and graphs were processed with Prism 9.

### Ethics statement for human pluripotent stem cells

This work employed human embryonic stem cell lines (WA09), the use of which was approved to the Zeltner lab by WiCell.

## Results

### Patterns of O-GlcNAcylation during symN development

**Figure 2:**
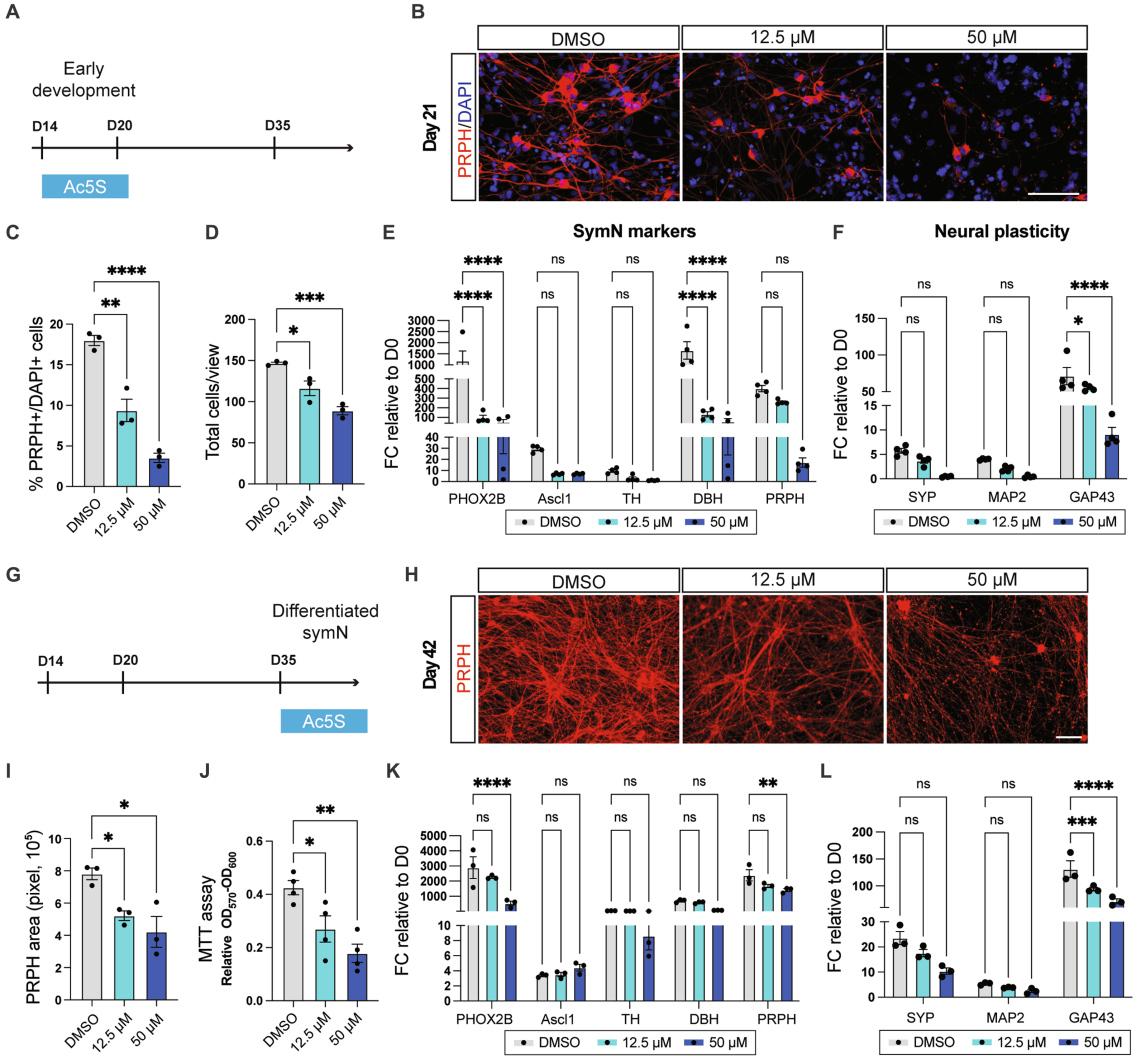
O-GlcNAcylation in symNs. **(A)** Schematic illustration of symN differentiation. **(B)** Cartoon of the O-GlcNAcylation mechanism with specific inhibitors used in this study. **(C)** RNA expression of *OGT* in hPSC-symNs by RT-qPCR. D0 indicates undifferentiated hPSCs at day 0 before induction of differentiation. One-way ANOVA followed by Dunnett’s multiple comparisons. *n* = 3 biological replicates. Each stage was compared to day 2. **(D)** RNA expression of *OGA* in hPSC-symNs by RT-qPCR. One-way ANOVA followed by Dunnett’s multiple comparisons. *n* = 3 biological replicates. Each stage was compared to day 2. **(E)** Left: RNA expression ratio of *Ogt*/*Oga* in mouse SCG by RT-qPCR. Unpaired *t* test. *n* = 4 biological replicates. Right: Re-analyzed bulk RNAseq data from mouse stellate ganglion (SG). Unpaired *t* test. *n* = 12 biological replicates. **(F)** Representative western blot analysis of protein O-GlcNAcylation in hPSC-symNs. Bands in each lane show O-GlcNAcylated proteins. **(G)** Quantification of expression from **(F)**. Each entire lane was quantified. One-way ANOVA followed by Dunnett’s multiple comparisons. *n* = 4 biological replicates. Each stage was compared to hPSC. **(H)** Representative western blot analysis of OGT/OGA expression in hPSC-symNs. **(I)** Quantification of expression ratio from **(H)**. One-way ANOVA followed by Dunnett’s multiple comparisons. *n* = 4 biological replicates. Each stage was compared to hPSC. **(J)** Representative immunofluorescent staining for O-GlcNAc and PRPH in hPSC-symNs. The dashed rectangle highlights the single neuron body as a representative picture for the intensity analysis in **(K)**. **(K)** Intensity distribution analysis from **(J)** shows highly overlayed O-GlcNAcylation signals and cell nucleus localization. White bar shows the analyzed fragment in the picture. Nuclear localization was referenced using DAPI. *n* = 3 biological replicates (three representative cells were quantified in each replicate). *p < 0.05, ***p < 0.001, ****p < 0.0001. Error bars represent SEM. Scale bars = 100 μm.

The directed postganglionic symN differentiation protocol we established previously starts with 10 days of neural crest cell (NCC) induction, to generate the precursor of postganglionic symNs (Ernsberger and Rohrer, 2018), followed by 4 days of sympathetic neuroblast (symNblast) spheroid induction in 3D culture. Spheroids are dissociated and replated for final symN differentiation (Wu and Zeltner, 2020; Figure 2A). Differentiated symNs using this protocol yield high purity and specificity, and express specific markers including *PHOX2B*, *ASCL1*, *TH*, *DBH*, *VMAT*, *ADR*s, and more (Wu and Zeltner, 2020; Wu et al., 2022) (TH staining is also shown in Supplementary Figure S1). In addition, differentiated day 10 NCCs can be frozen, which allows large scale experiments and ensures reproducible results from well characterized cells (Wong et al., 2017; Wu et al., 2022). Employing this protocol, we were able to track the pattern of O-GlcNAcylation throughout the symN developmental lifespan.

Regulation of O-GlcNAcylation relies on the activity of two enzymes, O-GlcNAc transferase (OGT) that catalyzes the addition of O-GlcNAc to proteins, and O-GlcNAcase (OGA) that catalyzes the removal of O-GlcNAc from proteins (Hart, 2019; Figure 2B). Therefore, we first examined whether O-GlcNAcylation is present in symN development by analyzing OGT/OGA RNA expressions during NCC induction (day 2–10), symNblast induction (day 14), and symN differentiation (day 35) using RT-qPCR. The result showed that OGT levels increased during NCC differentiation and dropped when the cells were driven toward symN fate, while OGA expressions showed the opposite pattern (Figures 2C,D). To confirm this finding, we analyzed *Ogt*/*Oga* RNA expressions from cultures of mouse superior cervical ganglion (SCG) symNs at E14.5, the timepoint when sympathetic ganglia just form (Hendry, 1977; Rothman et al., 1978; Rubin, 1985; Fagan et al., 1996; Armstrong et al., 2011). As what we observed in hPSC-derived symNs, mouse symNs also displayed higher *Oga* expression over *Ogt* (Figure 2E, left), which might imply reduced O-GlcNAcylation levels (Yang et al., 2020; Zuliani et al., 2021) in differentiated symNs. We also confirmed the higher *Oga* expression over *Ogt* in adult (16–19 weeks) mouse symNs by re-analyzing a published bulk RNAseq database (Bayles et al., 2018; Figure 2E, right). Using western blotting, we analyzed protein O-GlcNAcylation from day 0–35 of hPSC-derived symN differentiation. Indeed, O-GlcNAcylation rose at the NCC stage and dropped at the symN stage (Figures 2F,G). Accordingly, protein expressions of OGT over OGA were also decreased overtime (Figures 2H,I). These results suggest that O-GlcNAcylation levels reached their plateau at the early progenitor stage of symN development, which is in alignment with the current understanding that O-GlcNAcylation is largely present in stem cells and multipotent precursors (Sheikh et al., 2021).

Published studies suggest that O-GlcNAcylation takes place abundantly in the nucleus (Hart and Akimoto, 2009). Thus, we next investigated the distribution of O-GlcNAcylation in hPSC-derived symNs using immunostaining. We observed solid O-GlcNAcylation signals among all peripherin+ (PRPH, a pan peripheral neuron marker) symNs, especially in the nucleus (Figures 2J,K). This was further confirmed using other two O-GlcNAc antibodies, CTD110.6 and RL2 and co-staining with TH (Supplementary Figure S1A). Together, our data reveal that, like in other well-studied cell types, O-GlcNAcylation is present in symNs and it is finely regulated during development.

### O-GlcNAcylation participates in symN differentiation and maintenance

Although the level dropped, O-GlcNAcylation was stably present in differentiating symNs (Figure 2). Therefore, we next aimed to study the importance of O-GlcNAcylation in maintenance and maturation of symNs. To assess that, we treated symNs with OGT inhibitor Ac-5SGlcNAc (Ac5S, Figure 2B) to suppress O-GlcNAcylation.

**Figure 3:**
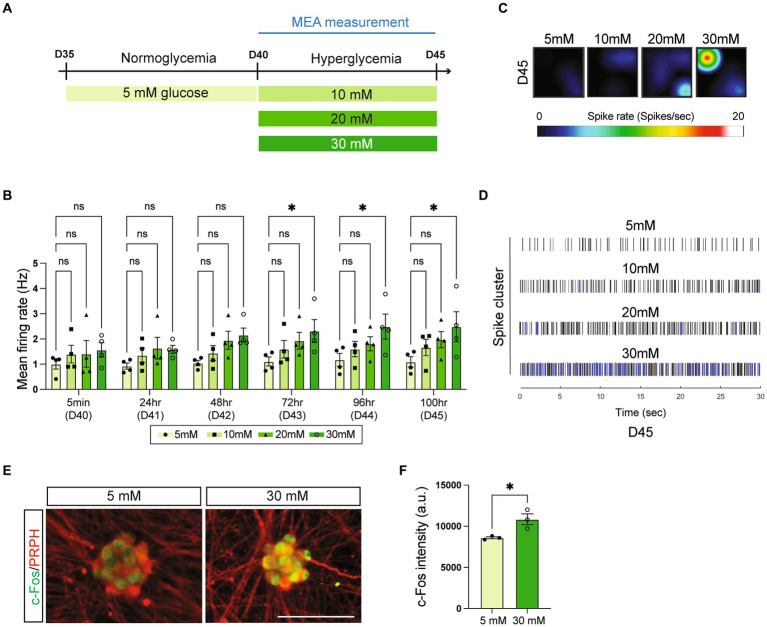
O-GlcNAcylation is crucial for symN differentiation and survival. **(A)** Schematic illustration of Ac5S treatment at early stages of the symN differentiation. **(B)** Representative immunofluorescent staining for PRPH on day 21 upon control DMSO or Ac5S treatment. **(C)** Quantification of PRPH positive cells on day 21. One-way ANOVA followed by Dunnett’s multiple comparisons. *n* = 3 biological replicates. **(D)** Quantification of DAPI positive total cells on day 21. One-way ANOVA followed by Dunnett’s multiple comparisons. *n* = 3 biological replicates. **(E)** RNA expression of symN markers on day 21 by RT-qPCR. Two-way ANOVA followed by Dunnett’s multiple comparisons. *n* = 4 biological replicates. **(F)** RNA expression of neural plasticity markers on day 21 by RT-qPCR. Two-way ANOVA followed by Dunnett’s multiple comparisons. *n* = 4 biological replicates. **(G)** Schematic illustration of Ac5S treatment at late stages of symN differentiation. **(H)** Representative immunofluorescent staining for PRPH on day 42. **(I)** Quantification of PRPH positive area on day 42. One-way ANOVA followed by Dunnett’s multiple comparisons. *n* = 3 biological replicates. **(J)** MTT assay for symN survival on day 42. One-way ANOVA followed by Dunnett’s multiple comparisons. *n* = 4 biological replicates. **(K)** RNA expression of symN markers on day 42 by RT-qPCR. Two-way ANOVA followed by Dunnett’s multiple comparisons. *n* = 3 biological replicates. **(L)** RNA expression of neural plasticity markers on day 42 by RT-qPCR. Two-way ANOVA followed by Dunnett’s multiple comparisons. *n* = 3 biological replicates. *p < 0.05, **p < 0.01, ***p < 0.001, ****p < 0.0001. FC, fold change. Error bars represent SEM. Scale bars = 100 μm.

We first treated Ac5S at low (12.5 μM) and high (50 μM) dosages on day 14–21, the first week of symN differentiation (Figure 3A). On day 21, Ac5S treatment decreased PRPH+ symN numbers in a dose dependent manner (Figures 3B,C), suggesting insufficient neural differentiation. In addition, the total cell number decreased upon Ac5S treatment (Figure 3D), showing that the proliferation of progenitor cells might be affected as well. RT-qPCR analysis showed decreased expression of symN markers (*PHOX2B*, *ASCL1*, *TH*, *DBH*, *PRPH*, Figure 3E) and markers for neural plasticity (*SYP*, *MAP2*, *GAP43*, Figure 3F). The dose dependent O-GlcNAcylation inhibition by Ac5S was confirmed using western blot (Supplementary Figure S2A). To make sure the impaired symN development is a specific effect by O-GlcNAcylation inhibition, we treated day 14–21 symNs with another OGT inhibitor, OSMI-4 (2 μM, Supplementary Figure S2B). Similar to Ac5S treatment, impaired symN differentiation and gene expressions were found on day 21 (Supplementary Figures S2C–G). O-GlcNAcylation inhibition by OSMI-4 was confirmed using western blot (Supplementary Figure S2H). These results demonstrate that inhibition of O-GlcNAcylation impaired early symN growth/maintenance. In addition, we also sought to elevate O-GlcNAcylation in developing symNs by treating the neurons with OGA inhibitor Thiamet-G (TMG, 1 μM, Supplementary Figure S3A). As the result, we observed moderate but significant increase of PRPH+ symNs, while the total cell number remained unchanged (Supplementary Figures S3B–D), suggesting improved neurogenesis. Interestingly, however, both symN and neural plasticity markers were decreased after TMG treatment (Supplementary Figures S3E,F). Elevated O-GlcNAcylation level by TMG was confirmed using western blot (Supplementary Figure S3G). It seemed that although increasing O-GlcNAcylation in neural progenitors could facilitate the progress of neural differentiation, the identity and functionality of the neurons might be impaired. These results are in line with a previous study in cortical neuron differentiation (Parween et al., 2017), and supports the notion that O-GlcNAcylation level needs to be precisely controlled to ensure normal symN development.

Next, alternatively, Ac5S was given to symNs for 1 week after day 35, the timepoint when symN differentiation is finalized (Figure 3G). As a result, symN complexity decreased in a dose dependent manner on day 42 (Figures 3H,I). Similarly, we assessed cell viability using the MTT assay and showed that symN viability decreased after Ac5S treatment (Figure 3J), suggesting that O-GlcNAcylation is required for symN survival. Finally, symN markers and neural plasticity markers again decreased (except *ASCL1* at the high dosage) due to the late stage Ac5S treatment (Figures 3K,L). The inhibitory effect of Ac5S to O-GlcNAcylation level in day 35 symNs was confirmed using western blot (Supplementary Figure S4A). In sum, these results show that O-GlcNAcylation is a crucial PTM for symNs, that governs both symN differentiation and maintenance.

### *In vitro* hyperglycemia model reveals symN hyperactivity

Next, we sought to study the change of symN O-GlcNAcylation in metabolic diseases. Considering the ubiquitous role of O-GlcNAcylation in sensing glucose levels in multiple organs in the body, we chose hyperglycemic toxicity in diabetes as our model system. As a nutrient and stress sensor, increasing O-GlcNAcylation has been linked to insulin resistance and to hyperglycemia-induced glucose toxicity (Ma and Hart, 2013). Dysregulated O-GlcNAcylation in organs such as pancreas, liver, heart, kidney, adipose tissues, and vascular endothelial cells, under chronic hyperglycemia has been found responsible for the dysfunction of these organs (Issad and Kuo, 2008). Interestingly, as the nervous system that regulates the metabolisms of all these organs, whether and how O-GlcNAcylation affects symN functions in hyperglycemia remains unknown. Moreover, SNS hyperactivity was suggested as an early hallmark of diabetes (Perin et al., 2001; Tentolouris et al., 2008; Lambert et al., 2015). Previous studies have demonstrated that postganglionic symNs are a powerful driver in the pathologies of autonomic diseases and cardiovascular disorders (Larsen et al., 2016; Wu et al., 2022). Hyperglycemic toxicity has been modeled in tissue cultures of multiple cell types in *in vitro* systems to evaluate the pathology of these tissues in diabetes, including symNs (Campanucci et al., 2010; Damon, 2011; Erickson et al., 2013; Pekkurnaz et al., 2014; Rudchenko et al., 2014; Chandna et al., 2015; Masaki et al., 2020; Albert-Garay et al., 2022; Majd et al., 2022). Therefore, we hypothesized that intrinsic symN hyperactivity may contribute to the neuropathy in diabetes, and we aimed to establish an *in vitro* hyperglycemia model to study its correlation with O-GlcNAcylation.

Neurobasal medium, the classic neuron culture base media contains high levels of glucose (i.e., 25 mM), compared to the normal human blood level of glucose (i.e., 5 mM) (Pekkurnaz et al., 2014). To mimic the diabetic environment, we first preconditioned symNs on day 35 to 5 mM glucose for 5 days, and then switched to high glucose conditions (10, 20, and 30 mM) on day 40 (Figure 1A). The medium was changed every day during day 35–45 to ensure that the glucose levels remained constant. Using multielectrode array (MEA) analysis, we assessed symN activity after day 40 and found that all high glucose environments elevated symN activity in a dose dependent manner, although only the 30 mM glucose treated group from 72 h on of treatment was significant (Figure 1B). This result, recapitulates the nature of diabetes as a chronic disorder. Indeed, symN hyperactivity on day 45 with 30 mM glucose was the highest (Figures 1C,D), and thus was selected as the best condition for further characterization. Such hyperactivity was also confirmed by immunostaining using the neuron activity marker c-Fos (Figures 1E,F).

### High glucose impairs the ability of symN regeneration

**Figure 4:**
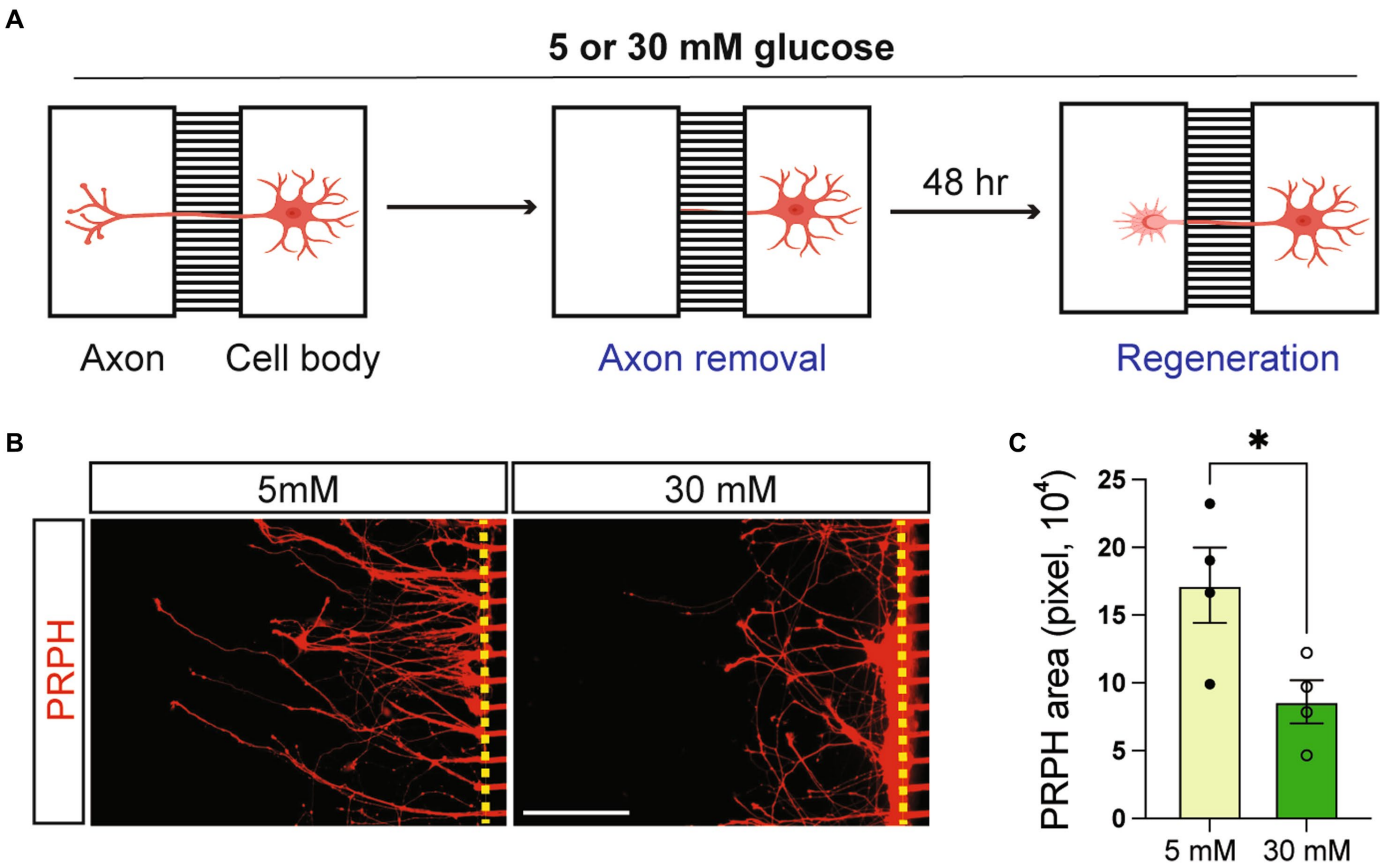
High glucose impairs symN regeneration. **(A)** Schematic illustration of symN regeneration assay using a microfluidic compartmental culture device. **(B)** Representative immunofluorescent staining for PRPH on the axon chamber 48 h after axon removal. **(C)** Quantification of PRPH positive area. Unpaired t test. *n* = 4 biological replicates. *p < 0.05. Error bars represent SEM. Scale bar = 100 μm.

Impaired PNS neuron regeneration has been discovered in patients and animal models of diabetes (Kennedy and Zochodne, 2005; Pham et al., 2018). To investigate whether our model recapitulates this feature, we performed a neuron regeneration assay using a microfluidic compartmental culture device. Axons of symNs that had undergone 5 to 5 mM or 5 to 30 mM transition of glucose conditions, were cut and allowed to regrow in the same conditions (Figure 4A). After 48 h, symNs were stained with the PRPH antibody in order to recognize the regenerated axons. We found that axons in 30 mM glucose showed lower levels of regeneration compared to the ones in 5 mM glucose (Figures 4B,C). This finding demonstrates that symN axon regeneration is hindered in high glucose conditions and may recapitulate the PNS defect found in diabetic patients.

### Increased O-GlcNAcylation mediates hyperglycemic symN hyperactivity

**Figure 5:**
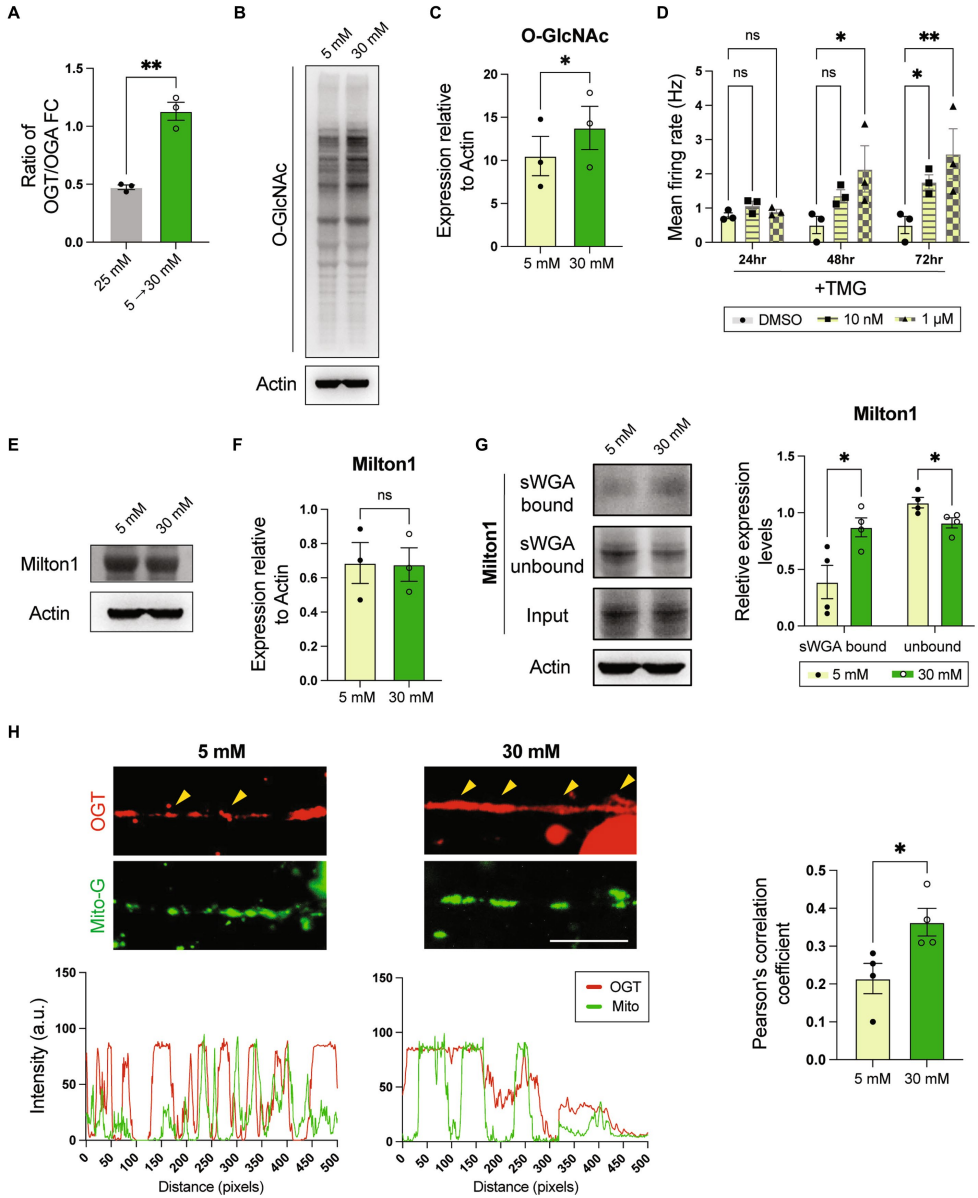
High glucose environment enhances O-GlcNAcylation that hyperactivates symNs. **(A)** RNA expression ratio of *OGT*/*OGA*. Unpaired *t* test. *n* = 3 biological replicates. **(B)** Representative western blot analysis for O-GlcNAc. **(C)** Quantification of O-GlcNAc level. Paired student *t*-test. *n* = 3 biological replicates. **(D)** MEA analysis of symN activity overtime after TMG treatments. Two-way ANOVA followed by Dunnett’s multiple comparisons. *n* = 3 biological replicates. **(E)** Representative western blot analysis for Milton. **(F)** Quantification of Milton expression. Unpaired student *t*-test. *n* = 3 biological replicates. **(G)** Pull-down assay. Cell lysates were pulled down with sWGA, and immunoblotted with Milton1 antibody. Multiple *t* test. *n* = 4 biological replicates. **(H)** Top: Immunostaining of OGT and mitochondrial labeling by MitoView mitochondria dye. Scale bar = 50 μm. Yellow arrows highlight the co-localization. Bottom: Distribution of signal intensity from pictures on the top. *p < 0.05, **p < 0.01. Error bars represent SEM.

With the model established, we next sought to investigate the roles of O-GlcNAcylation in symN pathology in high glucose as a nutrient sensor. RT-qPCR showed that the *OGT*/*OGA* ratio increased in symNs cultured in the diabetes-mimicking environment (5–30 mM transition of glucose, as established in Figure 1), even compared to the regular consistent 25 mM glucose condition (Figure 5A), suggesting that O-GlcNAcylation levels may be elevated in this model. Indeed, O-GlcNAcylated protein level was significantly increased after the high glucose transition (Figures 5B,C). To test whether increased O-GlcNAcylation is responsible for symN hyperactivity, we treated day 40 pre-conditioned neurons with TMG (Figure 2B). We found that TMG hyperactivates symNs in a dose-dependent manner, 72 h after treatment (Figure 5D), suggesting that increased O-GlcNAcylation causes symN hyperactivity. The effect of TMG on O-GlcNAcylation induction in preconditioned symNs was confirmed using western blot (Supplementary Figure S5A).

A previous study using a similar strategy, in which cultured rat hippocampal neurons were pre-conditioned in 5 mM glucose, and challenged with 30 mM glucose showed that the trafficking kinesin protein Milton is highly O-GlcNAcylated, which recruits OGT to the mitochondria and paralyzes mitochondrial movement (Pekkurnaz et al., 2014). In our model, western blot analysis showed that Milton expression itself was not different in 5–30 mM glucose transition compared to 5 to 5 mM (Figures 5E,F). In contrast, sWGA pull-down assay showed higher O-GlcNAcylated Milton level in high glucose (Figure 5G), suggesting that hyperglycemia also affects Milton function in symNs. To test whether impaired mitochondrial movement is also involved in high glucose-induced symN hyperactivity, we compared the localization of OGT and mitochondria by combining immunostaining and a mitochondrial tracer MitoView^™^ Green. The result showed that OGT and mitochondria are more co-localized in symNs in high glucose (yellow arrows, Figure 5H). In conclusion, increased O-GlcNAcylation and the involvement of mitochondrial defects might be responsible for high glucose induced symN hyperactivity.

### O-GlcNAc inhibition rescues hyperglycemia-induced symN hyperactivity

**Figure 6:**
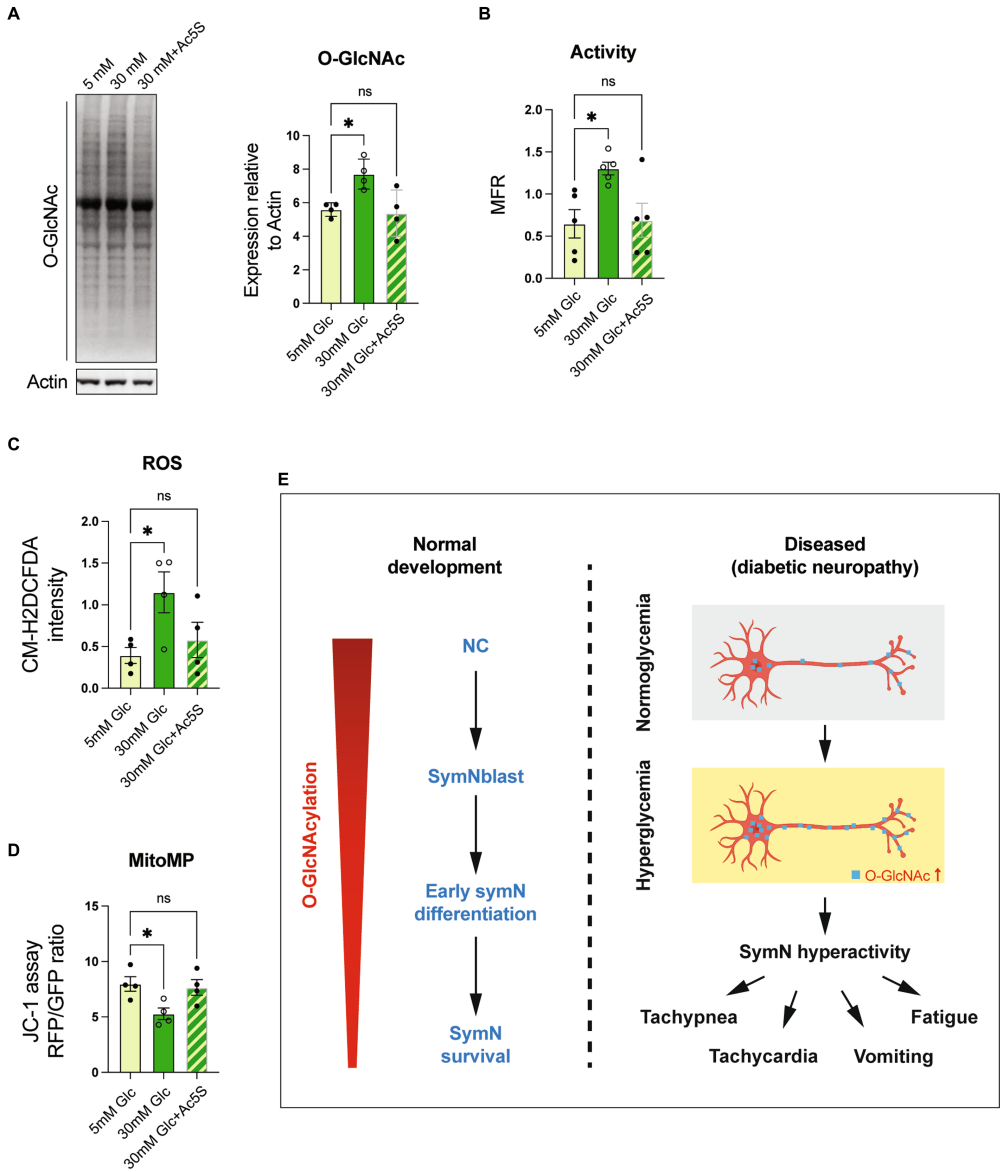
O-GlcNAcylation inhibition rescues symN hyperactivity in high glucose. **(A)** Western blot analysis for O-GlcNAc. One-way ANOVA followed by Dunnett’s multiple comparisons. *n* = 4 biological replicates. **(B)** MEA analysis of symN activity overtime after Ac5S treatment. One-way ANOVA followed by Dunnett’s multiple comparisons. *n* = 5 biological replicates. **(C)** ROS level of symNs assessed by CM-H2DCFDA. One-way ANOVA followed by Dunnett’s multiple comparisons. *n* = 5 biological replicates. **(D)** Mitochondrial membrane potential of symNs assessed by JC-1 assay. One-way ANOVA followed by Dunnett’s multiple comparisons. *n* = 4 biological replicates. **(E)** Summary of the roles of O-GlcNAcylation in symNs. Left: During normal symN development, O-GlcNAcylation governs neural crest (NC) induction, sympathetic neural blast (symNblast) fate determination, symN growth and survival. The level of O-GlcNAcylation decreases gradually along with symN differentiation, but still remains and is important for differentiated symNs. Right: In our *in vitro* model that mimics hyperglycemia in T2DM patients, O-GlcNAcylation increases, which hyperactivates symNs that might contribute to the metabolic and cardiovascular symptoms in the patients. *p < 0.05, Error bars represent SEM.

Finally, we examined if O-GlcNAcylation inhibition can rescue symN hyperactivity in the model of hyperglycemia (Figure 1). Along with 30 mM glucose, we treated preconditioned symNs with 12.5 μM Ac5S to suppress high glucose induced O-GlcNAcylation. As expected, 5–30 mM glucose transition with Ac5S dramatically suppressed O-GlcNAcylation, which was induced by 5–30 mM glucose transition (Figure 6A), and rescued symN hyperactivity (Figure 6B) induced by high glucose, yet it did not affect symN survival/health (Supplementary Figure S6A), suggesting that the rescue effect of Ac5S does not result from cell toxicity. We further looked for additional indications of the rescuing effect of O-GlcNAcylation inhibition in diabetic symNs. A recent study has shown high glucose toxicity in hPSC-derived Schwann cells (SCs) as a part of the peripheral diabetic neuropathy (Majd et al., 2022). Accordingly, it is well studied that hyperglycemia leads to cell stress in the nervous system (Edwards et al., 2008; Campanucci et al., 2010; Verrotti et al., 2014; Luna et al., 2021). Together with the finding of mitochondrial defects in published (Pekkurnaz et al., 2014) and our model systems (Figure 5H), we compared the cell stress level using (Hart, 2019) the ROS indicator CM-H2DCFDA, and (Sheikh et al., 2021) mitochondrial probe JC-1 to monitor mitochondrial membrane potential. We found that high glucose induced ROS accumulation and impaired mitochondrial polarization in symNs, which were rescued by Ac5S treatment at low dosage (Figures 6C,D). These results show that O-GlcNAcylation regulation might be a novel, future avenue for diabetes treatment via normalizing symN activity.

## Discussion

O-GlcNAcylation is a ubiquitous PTM, which has been shown indispensable in multiple organs in the body to support normal biological functions. O-GlcNAcylation governs differentiation and maturation of cells in the heart, skeletal muscles, bones, skin, fat tissues, and the brain (Sheikh et al., 2021). In β-cells (a cell type in the islet of the pancreas), O-GlcNAcylation of specific proteins regulates insulin secretion (Wu et al., 2017). In skeletal muscle and adipose tissue, increasing O-GlcNAcylation level leads to insulin resistance, and therefore is linked to glucose uptake and metabolism in these tissues (Lambert et al., 2018; Morino and Maegawa, 2021). In liver, O-GlcNAcylation controls gluconeogenesis mediated by insulin (Zhang et al., 2014).

The SNS regulates the functions of a wide range of organs in the body. The SNS is the key mediator of the baroreflex response that maintains blood pressure and homeostasis (Scott-Solomon et al., 2021). Activation of the SNS reduces insulin release and induces glucagon secretion and blood glucose by innervating the islet of the pancreas (Hampton et al., 2022). In skeletal muscle, SNS activity controls glycogenolysis, ionic transport, and contractility (Roatta and Farina, 2010). In adipose tissues, SNS is important for energy balance by regulating glucose uptake and lipolysis, and SNS even dictates the adipose transition to white or brown adipocyte (Munzberg et al., 2021). All the regulatory functions are achieved through catecholamine release (Ernsberger and Rohrer, 2018; Scott-Solomon et al., 2021).

A growing number of studies have shown that postganglionic symNs alone are a powerful driver to affect body homeostasis. For instance, Larsen et al. used a co-culture system of cardiac sympathetic stellate neurons and ventricular myocytes from rats and showed that symNs from hypertensive rats can induce a hypertensive state in healthy cardiac cells. Likewise, when hypertensive myocytes are co-cultured with healthy symNs, the cardiac functions of hypertensive myocytes are improved (Larsen et al., 2016). Recently, we studied phenotypes of symNs derived from patients of familial dysautonomia (FD), a genetic autonomic disorder, in which patients suffer from cardiovascular symptoms such as vomiting crisis and hypertension (Bar-Aluma, 1993). Using the symN protocol we established (Wu and Zeltner, 2020), we discovered intrinsic symN hyperactivity and increased NE secretion, which can affect cardiomyocyte beating in FD symNs (Wu et al., 2022). Accordingly, Winbo et al. also reported intrinsic hyperactivity in adrenergic symNs derived from patients of long QT syndrome type 1 (LQT1), a congenital cardiac syndrome (Winbo et al., 2021). In addition, studies also showed that symN activity can be regulated by its target tissues and the peripheral environments. For example, hyperinsulinemia, which is associated with insulin resistance in T2DM may increase SNS activity that worsens the cardiovascular complication (Russo et al., 2021), and the direct effect of insulin on promoting postganglionic symN activity using cultured rat SCG symNs has been demonstrated (Wolinsky et al., 1985).

This evidence prompted us to investigate the hypothesis that O-GlcNAcylation also regulates organ metabolism through regulating SNS function, and we were particularly interested in the roles it may play in postganglionic symNs. As expected, O-GlcNAcylation was found throughout the entire lifespan of symN differentiation, and the fact that its level was the highest during NCC differentiation coincides with previous studies in the brain, which suggest that high O-GlcNAcylation level maintains early progenitor identities (Sheikh et al., 2021; Figure 2). Furthermore, we found that although the level dropped, O-GlcNAcylation was still required to ensure symN growth and maintenance (Figure 3), which is similar to a previous study in sensory neurons using a sensory specific OGT knockout mouse model (Su and Schwarz, 2017).

A variety of tissues targeted by symNs are reported defective due to dysregulated O-GlcNAcylation in diabetes. For instance, eNOS activation in endothelial cells from patients with diabetes is impaired (Masaki et al., 2020). In the diabetic heart, increased O-GlcNAcylation activates CaMKII, which causes cardiac dysfunction such as arrhythmias and impaired calcium handling and cardiac cell death (Erickson et al., 2013; Ducheix et al., 2018; Prakoso et al., 2022). Increased O-GlcNAcylation also leads to β-cell failure (Pathak et al., 2008) and hyperinsulinemia (Tang et al., 2000), muscle atrophy (Lambert et al., 2018), kidney starvation (Sugahara et al., 2019), and more (Morino and Maegawa, 2021). In contrast, symNs regulate the functions of all these organs above, and yet it is still unknown how O-GlcNAcylation levels affect symNs in diabetes and other metabolic peripheral neuropathies. Thus, we sought to establish an *in vitro* hyperglycemia model in hPSC-symN culture to address this question.

Previous studies have demonstrated that the hyperglycemic condition can be reproduced *in vitro* in rat hippocampal neurons (Pekkurnaz et al., 2014), cardiomyocytes (Erickson et al., 2013), and retinal cells (Albert-Garay et al., 2022), human venous endothelial cells (Masaki et al., 2020), hPSC-SCs (Majd et al., 2022), and primary symNs (Campanucci et al., 2010; Damon, 2011; Rudchenko et al., 2014; Chandna et al., 2015). Semra et al. (2004) used 12 weeks old rat symN culture and found that high glucose (50–100 mM) causes symN stress and impairs neurite development and neuron survival. Damon (2011) co-cultured neonatal rat symNs with vascular smooth muscle cells and confirmed increased NE secretion in high glucose (25 mM). In our hands, by preconditioning symNs in culture medium containing 5 mM glucose and switching to 30 mM glucose, we observed symN hyperactivity accompanied by increased cell stress (Figures 1, 6C,D). We also demonstrated that increased O-GlcNAcylation in response to high glucose transition caused symN hyperactivity (Figures 5A–D), which was reversed by O-GlcNAcylation inhibition (Figures 6A–D). Studies in diabetic cells have shown increased O-GlcNAcylation in mitochondria (Pekkurnaz et al., 2014; Banerjee et al., 2015). Our results also confirmed impaired mitochondrial movement mediated by Milton in diabetic symNs (Figures 5E–H), in alignment with the results Pekkurnaz et al. (2014) found in diabetic rat hippocampal neurons. Whether the impaired mitochondrial function contributes to symN hyperactivity needs further investigation in the future.

Type II diabetes (T2D) comprises over 90% of the diabetic population in the US (Olokoba et al., 2012). Unhealthy dietary patterns that lead to insulin resistance is one of the biggest risk factors for developing T2D. T2D is a complication that involves hyperglycemia and hyperinsulinemia (Thomas et al., 2019). Previous studies have established *in vitro* T2D models using heart organoids and primary neurons by culturing the cells in high glucose combined with high insulin [1.14 nM (Lewis-Israeli et al., 2021) to 700 nM (Griggs et al., 2021)] conditions. The B27 supplement contains about 60 nM insulin, which is higher than the average level in the body [about 170 pM (Lewis-Israeli et al., 2021)]. Although the model we established here mainly focuses on the broader impact of O-GlcNAcylation in diabetic symNs by modeling hyperglycemia, the use of B27 in our culture media may imply that our hyperglycemic condition is an environment that resembles T2D more.

Lastly, SNS neuropathy at the early stage of diabetes is defined by increased symN activity and neurotransmitter release without neuronal loss, yet in prolonged diabetic complications, symN degeneration characterized by swollen symN axon terminals, reduced neurotransmitter release, and neuronal loss was observed, which is known as DAN (Schmidt, 1996, 2002a,b; Vinik et al., 2003). It would be an important next step to test if our hPSC-symN model can reproduce this long-term defect, in order to get a better understanding of sympathetic neuropathology in diabetes. In fact, substantial work by Campanucci et al. (2010) has demonstrated the impaired synaptic transmission in symN ganglia in diabetic mice and primary symN models, which may contribute to the progress of diabetic autonomic neuropathy (Rudchenko et al., 2014; Chandna et al., 2015). Using a streptozotocin-induced diabetic mouse model and low (5 mM) to high (25 mM) glucose transition in primary symN cultures, they showed impaired sympathetic neural transmission as well as decreased responsiveness after stimulation. This was found to be due to deactivated nicotinic acetylcholine receptors (nAChRs) in their relatively long term measurement (after 1 week to up to months) (Campanucci et al., 2010). This was subsequently found mediated by the receptor for advanced glycation end products (RAGE) (Chandna et al., 2015). Noticeably, RAGE activation and augmented O-GlcNAcylation can both be triggered by ROS (Lima et al., 2012). As a glucose and stress sensor, O-GlcNAcylation can also be induced by AGE (Li et al., 2007). Diabetic autonomic neuropathy in diabetic patients is a symptom that usually takes years to develop. Here, in our human symN model, we recapitulated the early symN hyperactivity under high glucose conditions (Figure 1), and we have confirmed that our differentiated healthy symNs can be maintained for over 100 days (data not shown). By combining the pioneering work by Campanucci et al. (2010) and our human-based hyperglycemia model, we may be able to establish the full spectrum of diabetic symN pathology and evaluate the effects of O-GlcNAcylation dysregulation in long-term conditions.

Together, our study highlights the crucial role of O-GlcNAcylation levels in inducing NC differentiation, initiating symN fate determination, facilitating symN growth, and supporting symN survival (Figure 6E, left). In addition, upregulation of O-GlcNAcylation due to the increased glucose concentration may hyperactivate symNs, which may worsen the defects observed in the downstream target tissues, and synergistically lead to the clinical symptoms found in the patients (Figure 6E, right).

## Data availability statement

The original contributions presented in the study are included in the article/Supplementary material, further inquiries can be directed to the corresponding author.

## Ethics statement

The studies involving human participants were reviewed and approved by WiCell. The patients/participants provided their written informed consent to participate in this study. The animal study was reviewed and approved by Institutional Animal Care and Use Committee University of Georgia.

## Author contributions

H-FW conceived, conducted and analyzed experiments, and wrote the manuscript. C-WH provided technical supports, conducted and analyzed western blot experiments, and wrote the manuscript. JA performed repeats of symN experiments. H-XL supervised the use of mouse tissues and edited the manuscript. GH supervised the experimental design of O-GlcNAc experiments and edited the manuscript. NZ lead the study, provided financial and administrative support, edited, and approved the manuscript. All authors contributed to the article and approved the submitted version.

## Funding

This work was funded by NIH/NINDS 1R01NS114567-01A1 to NZ, by R01DK61671 to GH and by the Georgia Research Alliance.

## Conflict of interest

GH receives a share of royalty received on sales of the CTD 110.6 antibody, managed by Johns Hopkins University.

The remaining authors declare that the research was conducted in the absence of any commercial or financial relationships that could be construed as a potential conflict of interest.

## Publisher’s note

All claims expressed in this article are solely those of the authors and do not necessarily represent those of their affiliated organizations, or those of the publisher, the editors and the reviewers. Any product that may be evaluated in this article, or claim that may be made by its manufacturer, is not guaranteed or endorsed by the publisher.
