# Supplementary material for: O-GlcNAcylation is crucial for sympathetic neuron development, maintenance, functionality and contributes to peripheral neuropathy
Source: Front Neurosci. 2023 May 5;17:1137847. doi: 10.3389/fnins.2023.1137847 (PMC10203903; doi:10.3389/fnins.2023.1137847)
Supplement: Supplementary file 1 [file Data_Sheet_1.PDF]

Supplementary Material

O-GlcNAcylation is crucial for sympathetic neuron development, maintenance, functionality and contributes to peripheral neuropathy

Authors and Affiliation

Hsueh-Fu Wu[#,1,2], Chia-Wei Huang[#,2,3], Jennifer Art[1,4], Hong-Xiang Liu[5], Gerald W. Hart[2,3] and Nadja Zeltner[1,2,6*]

[1]Center for Molecular Medicine, University of Georgia, Athens GA, USA

[2]Department of Biochemistry and Molecular Biology, University of Georgia, Athens GA, USA

[3]Complex Carbohydrate Research Center, University of Georgia, Athens GA, USA

[4]Neuroscience Program, Biomedical and Translational Sciences Institute, University of Georgia, Athens GA, USA

[5]Regenerative Bioscience Center, Department of Animal and Dairy Science, College of Agricultural and Environmental Sciences, University of Georgia, Athens, GA, USA

[6]Department of Cellular Biology, University of Georgia, Athens GA, USA

[#]Contributed evenly

***correspondence:**

Prof. Nadja Zeltner

nadja.zeltner@uga.edu

1 Supplementary Figures

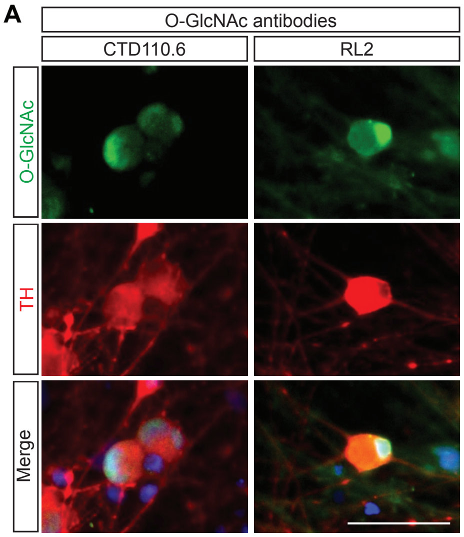

<u>**Supplementary figure 1**. Distribution of O-GlcNAcylation in symNs confirmed by multiple antibodies</u>

(A) Representative immunofluorescent staining for symN specific marker TH, and O-GlcNAcylation using two antibodies (CTD110.6 and RL2). Scale bar=50 µm.

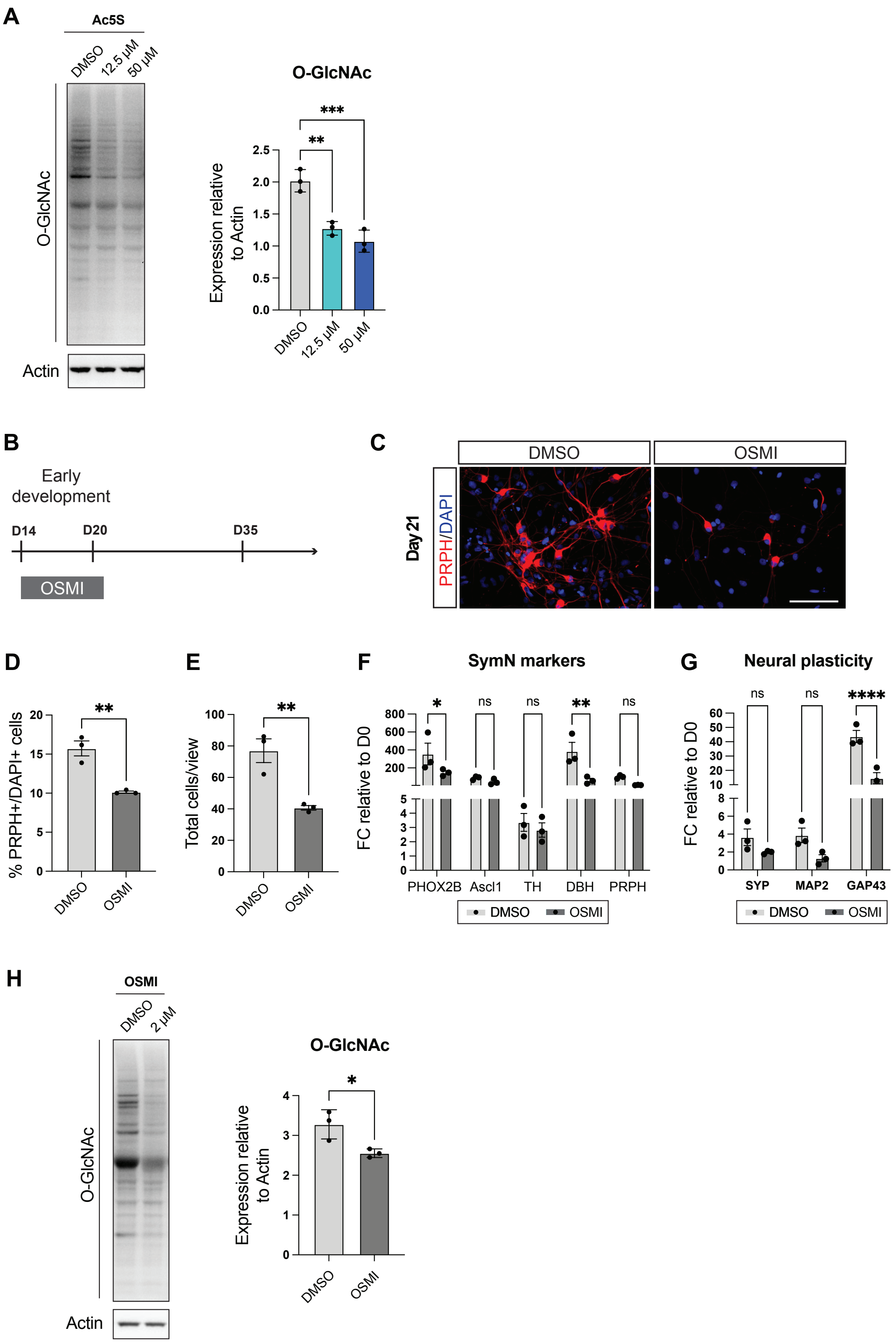

Supplementary figure 2. Impaired symN development due to suppressed O-GlcNAcylation using Ac5S/OSMI-4

(A) Western blot analysis for O-GlcNAcylation in day 15 symNs after Ac5S treatments for 24 hrs. One-way ANOVA followed by Dunnett's multiple comparisons. n=3 biological replicates. **(B)** Schematic illustration of OSMI-4 treatment at early stages of the symN differentiation. **(C)**

Representative immunofluorescent staining for PRPH on day 21 upon control DMSO or OSMI-4 (2 µM) treatment. **(D)** Quantification of PRPH positive cells on day 21. One-way ANOVA followed by Dunnett's multiple comparisons. n=3 biological replicates. **(E)** Quantification of DAPI positive total cells on day 21. One-way ANOVA followed by Dunnett's multiple comparisons. n=3 biological replicates. **(F)** RNA expression of symN markers on day 21 by RT-qPCR. 2-way ANOVA followed by Dunnett's multiple comparisons. n=3 biological replicates. **(G)** RNA expression of neural plasticity markers on day 21 by RT-qPCR. 2-way ANOVA followed by Dunnett's multiple comparisons. n=3 biological replicates. **(H)** Western blot analysis for O-GlcNAcylation in day 15 symNs after OSMI-4 treatment for 24 hr. Unpaired student t-test. n=3 biological replicates. *p<0.05, **p<0.01, ***, p<0.001, ****P<0.0001. FC=fold change. Error bars represent SEM. Scale bars=100 µm.

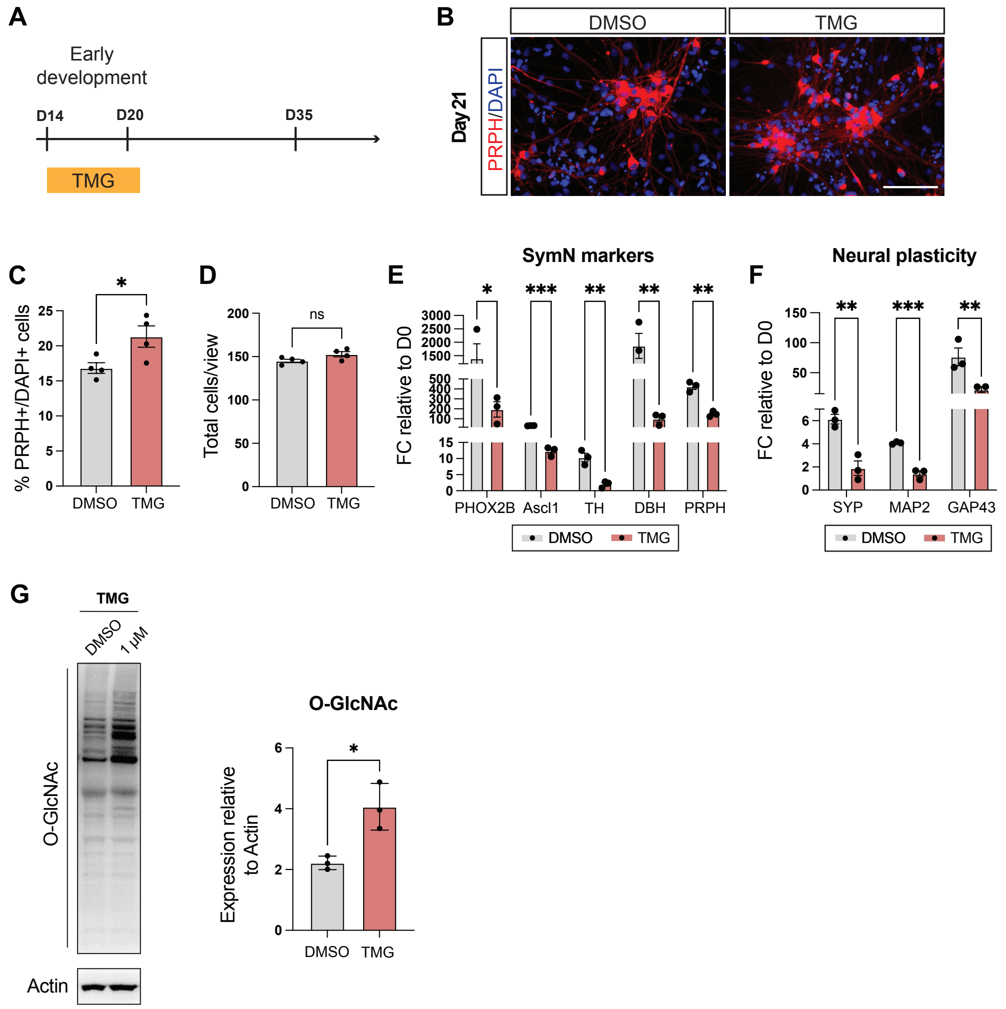

Supplementary figure 3. Excessive O-GlcNAcylation also impairs symN development

(A) Schematic illustration of TMG treatment at early stages of the symN differentiation. **(B)** Representative immunofluorescent staining for PRPH on day 21 upon control DMSO or TMG (1

µM) treatment. **(C)** Quantification of PRPH positive cells on day 21. Unpaired t test. n=4 biological replicates. **(D)** Quantification of DAPI positive total cells on day 21. Unpaired t test. n=4 biological replicates. **(E)** RNA expression of symN markers on day 21 by RT-qPCR. Multiple unpaired t test. n=3 biological replicates. **(F)** RNA expression of neural plasticity markers on day 21 by RT-qPCR. Multiple unpaired t test. n=3 biological replicates. **(G)** Western blot analysis for O-GlcNAcylation in day 15 symNs after TMG treatment for 24 hr. Unpaired student t-test. n=3 biological replicates. *p<0.05, **p<0.01, ***, p<0.001. FC=fold change. Error bars represent SEM. Scale bars=100 µm.

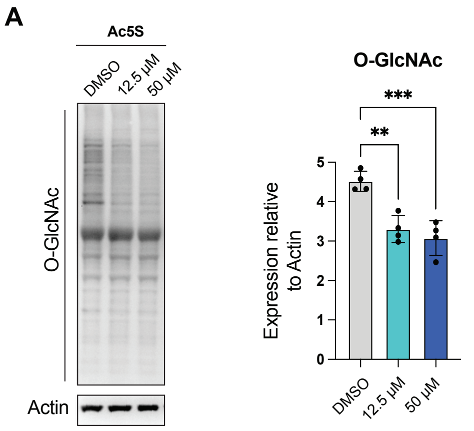

Supplementary figure 4. O-GlcNAcylation level can be suppressed by Ac5s in differentiated symNs

(A) Western blot analysis for O-GlcNAcylation in day 36 symNs after Ac5S treatments for 24 hr. One-way ANOVA followed by Dunnett's multiple comparisons. n=4 biological replicates. **p<0.01, ***, p<0.001. Error bars represent SEM.

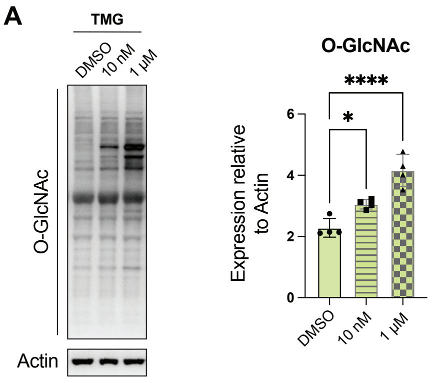

Supplementary figure 5. O-GlcNAcylation level is elevated by TMG in 5 mM glucose preconditioned symNs

(A) Western blot analysis for O-GlcNAcylation in day 41 5 mM glucose preconditioned symNs after TMG treatments for 24 hr. One-way ANOVA followed by Dunnett's multiple comparisons. n=4 biological replicates. *p<0.05, ****P<0.0001. Error bars represent SEM.

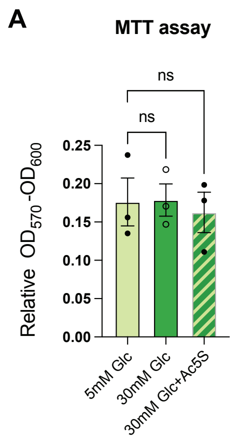

Supplementary figure 6. Ac5S treatment in addition to high glucose does not affect symN viability

(A) MTT assay for symN after high glucose and/or Ac5S treatments. One-way ANOVA followed by Dunnett's multiple comparisons. n=3 biological replicates.
